# Supplementary material for: Retrospective analysis of real-world data to evaluate actionability of a comprehensive molecular profiling panel in solid tumor tissue samples (REALM study)
Source: PLoS One. 2023 Sep 14;18(9):e0291495. doi: 10.1371/journal.pone.0291495 (PMC10501576; doi:10.1371/journal.pone.0291495)
Supplement: S1 Table — (DOCX) [file pone.0291495.s001.docx]

**Characteristics of patients with metastatic disease at F1CDx report in the evaluable population**

|  | **Lung cancer**  *N=136* | **Rare tumors^a^**  *N=39* | **Other tumors^b^**  *N=137* | **Total**  *N=312* |
| --- | --- | --- | --- | --- |
|  |  |  |  |  |
| **Demographics** | *N=136* | *N=39* | *N=137* | *N=312* |
| Age (years), mean±SD | 62.3 ± 10.3 | 52.6 ± 15.2 | 59.0 ± 13.3 | 58.9 ± 12.7 |
| Age ≥60 years | 80 (58.8) | 18 (54.7) | 75 (46.2) | 173 (55.5) |
| Male sex | 74 (54.4) | 23 (59.0) | 61 (44.5) | 158 (50.6) |
| **ECOG performance** | *N=133* | *N=38* | *N=123* | *N=294* |
| 0 | 32 (24.1) | 13 (34.2) | 41 (33.3) | 86 (29.3) |
| 1 | 76 (57.1) | 16 (42.1) | 60 (48.8) | 152 (51.7) |
| >1 | 25 (18.8) | 9 (23.7) | 22 (17.9) | 56 (19.0) |
| **At least one comorbidity*** | *N=130* | *N=37* | *N=120* | *N=287* |
|  | 62 (47.7) | 12 (32.4) | 68 (56.7) | 142 (49.5) |
| **Smoking status (at diagnosis)** | *N=135* | *N=16* | *N=85* | *N=236* |
| Former smoker | 57 (42.2) | 3 (18.8) | 26 (30.6) | 86 (36.4) |
| Smoker | 54 (40.0) | 3 (18.8) | 13 (15.3) | 70 (29.7) |
| Non-smoker ever | 24 (17.8) | 10 (62.5) | 46 (54.1) | 80 (33.9) |
| **At least one genetic test performed prior to F1CDx test** | *N=136* | *N=39* | *N=137* | *N=312* |
|  | 79 (58.1) | 6 (15.4) | 51 (37.2) | 136 (43.6) |

SD: standard variation

*Comorbidity: arterial hypertension, cardiovascular disease, dermatological disease, gastrointestinal disease, genital tract disease, infections, metabolic disease, neuromuscular disease, neuropsychiatric disease, ophthalmic disease, osteoarticular disease, other respiratory diseases, type I or II diabetes)

NOTE. Data presented as number (%) unless indicated otherwise

^a^ Sarcoma (n=13), thymic carcinoma (n=8), other rare tumors (n=18)

^b^ Biliary tract (n=18), skin (n=26), brain (n=8), bladder (n=27), breast (n=21), stomach (n=2), colorectal (n=5), pancreas (n=5), esophagus (n=3), liver (n=1), mouth (n=2), ovary (n=4), hail intestine (n=2), uterine (n=1), uterine cervix (n=1), unknown primary (n=11)

**Results of the F1CDx test in the evaluable population with metastatic disease**

|  | **Lung cancer**  *N=136* | **Rare tumors^a^**  *N=39* | | **Other tumors^b^**  *N=137* | | **Total**  *N=312* |  |
| --- | --- | --- | --- | --- | --- | --- | --- |
|  |  |  | |  | |  |  |
| **Genomic alterations** | N=136 | N=39 | | N=137 | | N=312 |  |
| Any genomic alterations identified | 136 (100.0) | 33 (84.6) | | 137 (100.0) | | 306 (98.1) |  |
| ]1-5] | 62 (45.6) | 22 (48.9) | | 67 (66.7) | | 151 (49.3) |  |
| ]5-10] | 59 (43.4) | 3 (27.7) | | 38 (9.1) | | 100 (32.7) |  |
| >10 | 6 (4.4) | 1 (6.6) | | 9 (3.0) | | 16 (5.2) |  |
| **Type of genomic alterations identified** | *N=136* | *N=33* | | *N=137* | | *N=306* |  |
| **Mutation or single-nucleotide variation** | | | | | | |  |
| Identification | 134 (98.5) | 30 (90.9) | | 123 (89.8) | | 287 (93.8) |  |
| Mean number ± SD | 3.7 ± 2.0 | 1.8 ± 0.8 | | 3.3 ± 2.6 | | 3.3 ± 2.3 |  |
| Median | 3.0 | 2.0 | | 3.0 | | 3.0 |  |
| Q1-Q3 | [2.0 , 5.0] | [1.0 , 2.0] | | [2.0 , 4.0] | | [2.0 , 4.0] |  |
| Range | (1.0,13.0) | (1.0,4.0) | | (1.0,19.0) | | (1.0,19.0) |  |
| **Insertion/deletion (indels)** | | | | | | |  |
| Identification | 45 (33.1) | 11 (33.3) | | 48 (35.0) | | 104 (34.0) |  |
| Mean number ± SD | 1.4 ± 0.6 | 1.4 ± 0.9 | | 1.6 ± 0.7 | | 1.5 ± 0.7 |  |
| Median | 1.0 | 1.0 | | 1.0 | | 1.0 |  |
| Q1-Q3 | [1.0 , 2.0] | [1.0 , 1.0] | | [1.0 , 2.0] | | [1.0 , 2.0] |  |
| Range | (1.0,3.0) | (1.0,4.0) | | (1.0,3.0) | | (1.0,4.0) |  |
| **Amplification or copy number variation** | | | | | | |  |
| Identification | 62 (45.6) | 10 (30.3) | | 56 (40.9) | | 128 (41.8) |  |
| Mean number ± SD | 2.8 ± 1.8 | 2.8 ± 2.3 | | 3.0 ± 1.8 | | 2.9 ± 1.8 |  |
| Median | 2.5 | 2.0 | | 3.0 | | 2.0 |  |
| Q1-Q3 | [1.0 , 3.8] | [1.3 , 2.8] | | [2.0 , 4.0] | | [1.0 , 4.0] |  |
| Range | (1.0,11.0) | (1.0,8.0) | | (1.0,9.0) | | (1.0,11.0) |  |
| **Rearrangement** | | | | | | |  |
| Identification | 7 (5.1) | 4 (12.1) | | 10 (7.3) | | 21 (6.9) |  |
| **Microsatellite status** | *N=130* | *N=38* | *N=117* | | *N=285* | | |
| Stable | 129 (99.2) | 38 (100.0) | 114 (97.4) | | 281 (98.6) | | |
| High | 1 (0.8) | 0 | 3 (2.6) | | 4 (1.4) | | |
| **Tumor mutation burden** (mutations/ megabase) | *N=129* | *N=38* | *N=119* | | *N=286* | | |
| Mean ± SD | 14.3 ± 12.7 | 4.0 ± 2.5 | | 11.3 ± 19.9 | | 11.7 ± 15.7 |  |
| ≤ 5 | 30 (23.3) | 28 (73.7) | | 61 (51.3) | | 119 (41.6) |  |
| [6-19] | 69 (53.5) | 10 (26.3) | | 45 (37.8) | | 124 (43.4) |  |
| ≥ 20 | 30 (23.3) | 0 | | 13 (10.9) | | 43 (15.0) |  |

SD, standard variation

NOTE. Data presented as number (%) unless indicated otherwise

^a^ Sarcoma (n=13), thymic carcinoma (n=8), other rare tumors (n=18)

^b^ Biliary tract (n=18), skin (n=26), brain (n=8), bladder (n=27), breast (n=21), stomach (n=2), colorectal (n=5), pancreas (n=5), esophagus (n=3), liver (n=1), mouth (n=2), ovary (n=4), hail intestine (n=2), uterine (n=1), uterine cervix (n=1), unknown primary (n=11)
