# Supplementary material for: Retrospective analysis of real-world data to evaluate actionability of a comprehensive molecular profiling panel in solid tumor tissue samples (REALM study)
Source: PLoS One. 2023 Sep 14;18(9):e0291495. doi: 10.1371/journal.pone.0291495 (PMC10501576; doi:10.1371/journal.pone.0291495)

**S2. Gene pathway groups:** occurrence of the 4 main classes of genomic alterations in genes belonging to 6 (+1) potentially targetable gene families/pathways (TK: tyrosine kinase, DDR: DNA damage repair, CC: cell cycle, PAM: PI3K-ATM-mTOR, RME: RAF-MEK-ERK, IE: immune evasion, OTH: others)


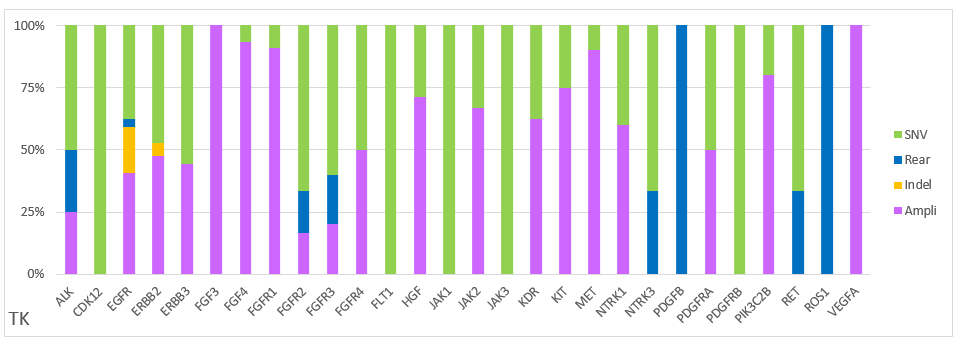


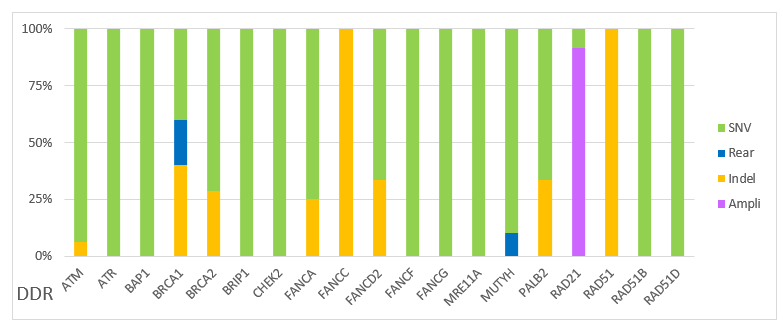


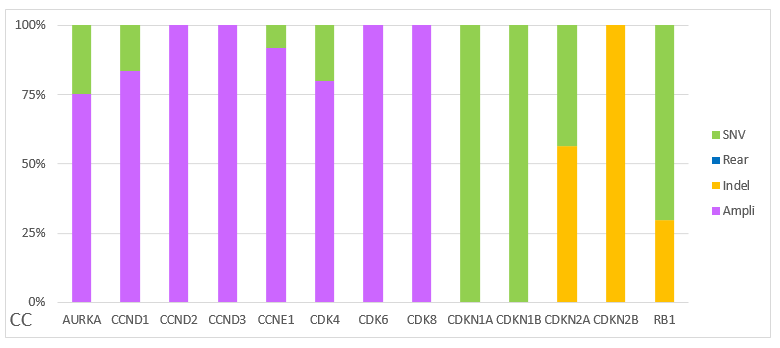


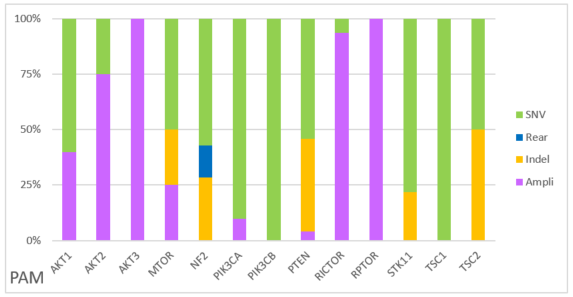


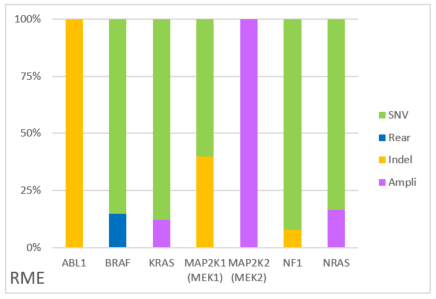


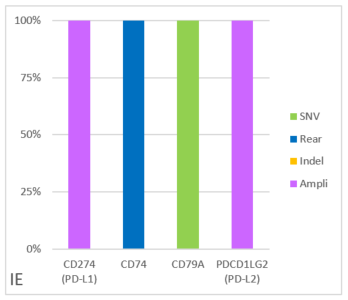


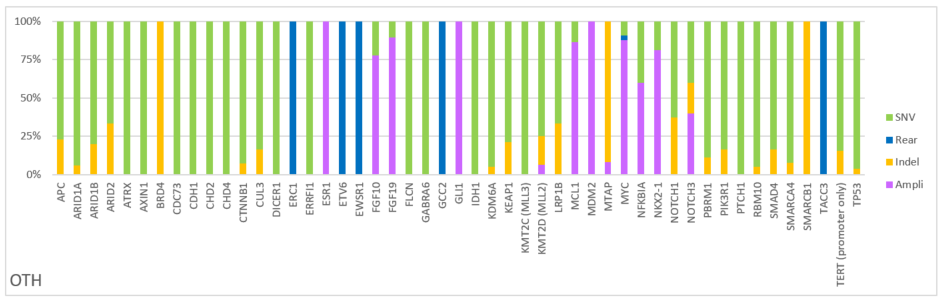

Supplement: S2 File — Occurrence of the 4 main classes of genomic alterations in genes belonging to 6 potentially targetable gene families/pathways (TK: tyrosine kinase, DDR: DNA damage repair, CC: cell cycle, PAM: PI3K-ATM-mTOR, RME: RAF-MEK-ERK, IE: immune evasion, OTH: others). (DOCX) [file pone.0291495.s005.docx]
